# Single-Cell RNA Sequencing Reveals the Cellular Landscape of *Longissimus Dorsi* in a Newborn Suhuai Pig

**DOI:** 10.3390/ijms25021204

**Published:** 2024-01-18

**Authors:** Wei Xiao, Nengjing Jiang, Zhengyu Ji, Mengru Ni, Zhaobo Zhang, Qingbo Zhao, Ruihua Huang, Pinghua Li, Liming Hou

**Affiliations:** 1 College of Animal Science and Technology, Nanjing Agricultural University, Nanjing 210095, China; weifortunately@gmail.com (W.X.); 18751997986@njau.edu.cn (N.J.); jzy604672583@gmail.com (Z.J.); nmr1999@163.com (M.N.); zhaobo1312@126.com (Z.Z.); zhaoqingbo@njau.edu.cn (Q.Z.); rhhuang@njau.edu.cn (R.H.); lipinghua718@njau.edu.cn (P.L.); 2 Institute of Swine Science, Nanjing Agricultural University, Nanjing 210095, China; 3 Key Laboratory of Pig Genetic Resources Evaluation and Utilization (Nanjing) of Ministry of Agriculture and Rural Affairs, Nanjing Agricultural University, Nanjing 210095, China; 4 Huai’an Academy, Nanjing Agricultural University, Huai’an 223001, China

**Keywords:** pig, *longissimus dorsi*, muscle, single-cell RNA sequencing, cellular atlas, fibro-adipogenic progenitors

## Abstract

The introduction of single-cell RNA sequencing (scRNA-seq) technology has spurred additional advancements in analyzing the cellular composition of tissues. The *longissimus dorsi* (LD) in pigs serves as the primary skeletal muscle for studying meat quality in the pig industry. However, the single-cell profile of porcine LD is still in its infancy stage. In this study, we profiled the transcriptomes of 16,018 cells in the LD of a newborn Suhuai pig at single-cell resolution. Subsequently, we constructed a cellular atlas of the LD, identifying 11 distinct cell populations, including endothelial cells (24.39%), myotubes (18.82%), fibro-adipogenic progenitors (FAPs, 18.11%), satellite cells (16.74%), myoblasts (3.99%), myocytes (5.74%), Schwann cells (3.81%), smooth muscle cells (3.22%), dendritic cells (2.99%), pericytes (1.86%), and neutrophils (0.33%). CellChat was employed to deduce the cell–cell interactions by evaluating the gene expression of receptor–ligand pairs across different cell types. The results show that FAPs and pericytes are the primary signal contributors in LD. In addition, we delineated the developmental trajectory of myogenic cells and examined alterations in the expression of various marker genes and molecular events throughout various stages of differentiation. Moreover, we found that FAPs can be divided into three subclusters (*NR2F2*-FAPs, *LPL*-FAPs, and *TNMD*-FAPs) according to their biological functions, suggesting that the FAPs could be associated with the differentiation of tendon cell. Taken together, we constructed the cellular atlas and cell communication network in LD of a newborn Suhuai pig, and analyzed the developmental trajectory of myogenic cells and the heterogeneity of FAPs subpopulation cells. This enhances our comprehension of the molecular features involved in skeletal muscle development and the meat quality control in pigs.

## 1. Introduction

With the increasing demand for high-quality pork, enhancing meat quality has become a prominent focal point in livestock husbandry research. The *longissimus dorsi* (LD) plays a crucial role in determining pork quality, and has been utilized to assess various meat quality traits, including meat color [1], flavor [2], tenderness [3], and others. The inherent cellular composition and physiological functions of meat endow its characteristics [4,5]. As a representative muscle model for assessing lean meat production and evaluating meat quality in pig breeding, the LD has been the focus of numerous studies, encompassing relevant data such as histological, genetic, transcriptomic, and epigenetic information [6,7].

Currently, single-cell RNA expression profiling is rapidly becoming an essential method for a diverse array of studies, including research on humans, animals, and plants. It allows for a more precise and rapid identification of rare and novel cells in tissues than ever before [8,9]. A recent study examined 483 samples from various tissues of the human body, constructing the cellular atlas and gene expression profiles of virtually all human tissues [10]. Analogous investigations have been carried out in mice, cattle, corn, Arabidopsis, and various other species [9,11,12,13,14]. Regarding scRNA-seq in porcine species, single-cell atlases and gene expression profiles of the skin [15], lungs [16], muscles [17], embryos [5], and peripheral blood [18] have been reported. A study has unveiled the transcriptional profiles of over 200,000 porcine cells across 20 tissues (excluding muscle) in pigs through scRNA-seq [19]. Recently, a study disclosed the single-cell profiles of LD from three distinct pig breeds (Wild boars, Laiwu pigs, and Duroc pigs), unveiling variations in cell populations, communication signaling networks, and skeletal muscle expression profiles among three different pig breeds [6]. These studies reveal heterogeneity in the physiological properties of LD among different pig breeds.

The Chinese Suhuai pig is a recently developed lean-type breed, comprised of 25% Chinese Huai pig lineage and 75% Large White lineage [20]. However, the cellular composition of LD remains unclear in newborn Suhuai pigs. In this study, we constructed the cellular atlas, explored the intercellular communication network, and delineated the developmental trajectory of myogenic cells as well as the heterogeneity of FAPs subpopulations in a 1-day-old newborn Suhuai boar using scRNA-seq. Our findings serve as a valuable resource for comprehending the molecular and cytological foundations of skeletal muscle development, along with the regulation of meat quality traits in pigs. 

## 2. Results

### 2.1. Identification the Cell Populations of LD of a 1-Day-Old Newborn Suhuai Boar

We conducted scRNA-seq on a mononuclear cell suspension isolated from the LD muscle of a 1-day-old newborn Suhuai boar. Following rigorous filtrations, 9508 cells were retained for subsequent analyses (Supplementary Figure S1A). The cells of LD were integrated and categorized into 13 clusters (clusters 0–12) through unsupervised clustering analysis (Supplementary Figure S1B,C).

**Figure 1:**
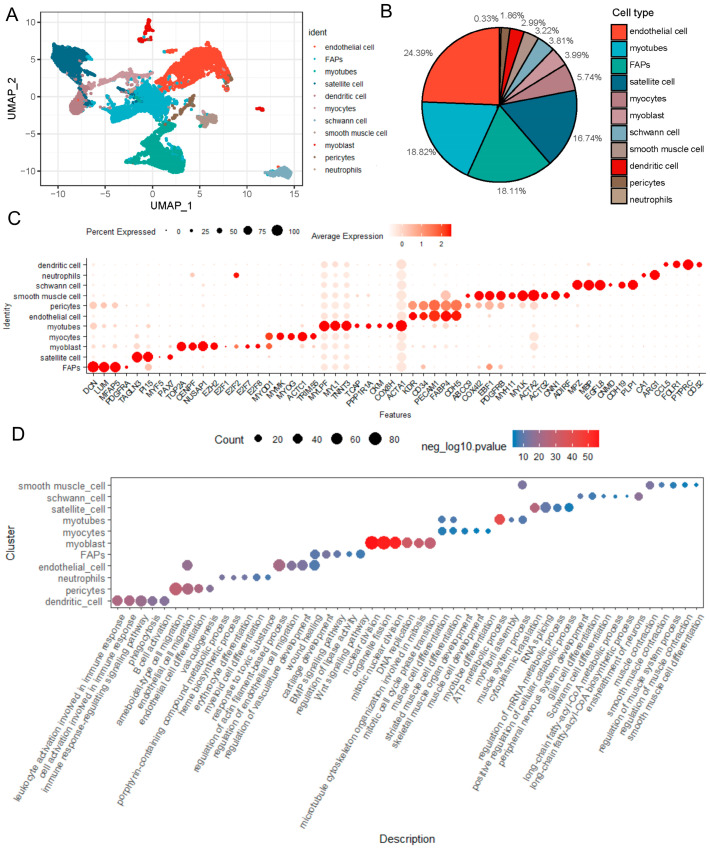
Identification of cell populations in LD of Suhuai pigs using scRNA-seq. (**A**) UMAP plot identified 11 cell clusters in LD of the newborn Suhuai pig using 10× Genomics scRNA-seq. (**B**) The proportional of cellular composition in LD as shown in A. (**C**) Dot plot of selected differentially expressed marker genes (*x*-axis) from Supplementary Table S1 for annotated different cell types (*y*-axis). The size of dot represents the percent of cell expressed the designated markers genes in certain cell types. The color depth of dot corresponds to the average relative gene expression in a certain cell type versus the other cell types. (**D**) Dot plot of selected enriched Gene Ontology terms (*x*-axis) from Supplementary Table S2 for each annotated different cell types (*y*-axis) shown in A. The size of dot corresponds to the count value for certain GO. The color depth of dot corresponds to the negative log10 *p*-value for certain GO.

For characterizing the properties of different cell clusters, we examined the differentially expressed genes (DEGs) in each cluster. Owing to the similarity in gene expression patterns observed (Supplementary Figure S1D), clusters 2 and 4, as well as clusters 3 and 6, were merged together. In total, we annotated 11 different clusters using both differential expression analysis, established marker genes from the existing literature, and computed the percentage distribution of these cell types (Figure 1A,B). As show in Figure 1B, we found that endothelial cells were the most abundant, comprising 24.39% of the total cell population in LD of a newborn Suhuai pig. Additionally, myotubes constituted 18.82%, FAPs accounted for 18.11%, satellite cells for 16.74%, and the remaining cell types collectively comprised less than 6%, with neutrophils being the least prevalent at 0.33%.

While numerous cellular marker genes have been documented, their expression levels may vary due to factors such as species, age, and health status. Consequently, we gathered recently reported cellular marker genes from various species, including humans, and profiled their expression levels in various cell populations of LD of newborn Suhuai pigs (Figure 1C). We noted that the classical marker genes *MYF5* and *PDGFRA* showed very low expression levels in satellite cells and FAPs, respectively. In addition, we found that genes associated with mitosis, namely DNA topoisomerase II α (*TOP2A*), centromere protein F gene (*CENPF*), and nucleolar and spindle-associated protein 1 gene (*NUSAP1*), were either specifically (*TOP2A*) or highly expressed (*CENPF* and *NUSAP1*) in myoblasts. Intriguingly, the epigenetic modification enzyme *EZH2* gene, a catalytic subunit of the Polycomb repressive complex 2 (*PRC2*), exhibited unique expression in myoblasts. In addition to *E2F2*, which was highly expressed in neutrophils, the other three cell cycle associated transcription factor *E2F* family genes *E2F1*, *E2F7* and *E2F8* were also uniquely expressed in myoblasts. Moreover, the expression of *PAX7* gradually decreased from satellite cells to myoblasts and myocytes, respectively, while the expression of *MYOD1* gradually increased from satellite cells to myoblasts and myocytes, respectively (Supplementary Table S1 and Figure 1C).

Next, we conducted the Gene Ontology (GO) analysis of the differentially expressed genes (DEGs) for each cell type (Figure 1D). Overall, 3649 biological processes terms were significantly enriched (Supplementary Table S2). We found that 11 cell types preferentially enriched selected different GOs, although some GOs were shared with different cell types. For instance, the dendritic cells were largely involved in the regulation of biological processes, such as immune response-regulating signaling pathway and B cell activation, etc. The endothelial cells were enriched mainly in endothelial cell migration, which belongs to one of the pericyte-enriched GOs. In addition, myoblasts were significantly enriched in biological processes related to nuclear division, DNA replication, cell cycles and mitosis-related GOs, which is consistent with the function of marker genes shown in Figure 1C. Furthermore, FAPs were enriched in wound healing, regulation of lipase activity, cartilage development, and BMP and Wnt signaling pathways (Figure 1D), suggesting its multi-directional differentiation potential may be governed by BMP and Wnt signaling pathways. In summary, these results revealed the cellular composition, differentially expressed marker genes, and distinct functions for various cell types in LD of newborn Suhuai pigs.

### 2.2. Analysis of Cell Communication in the LD of the Newborn Suhuai Pig by CellChat

**Figure 2:**
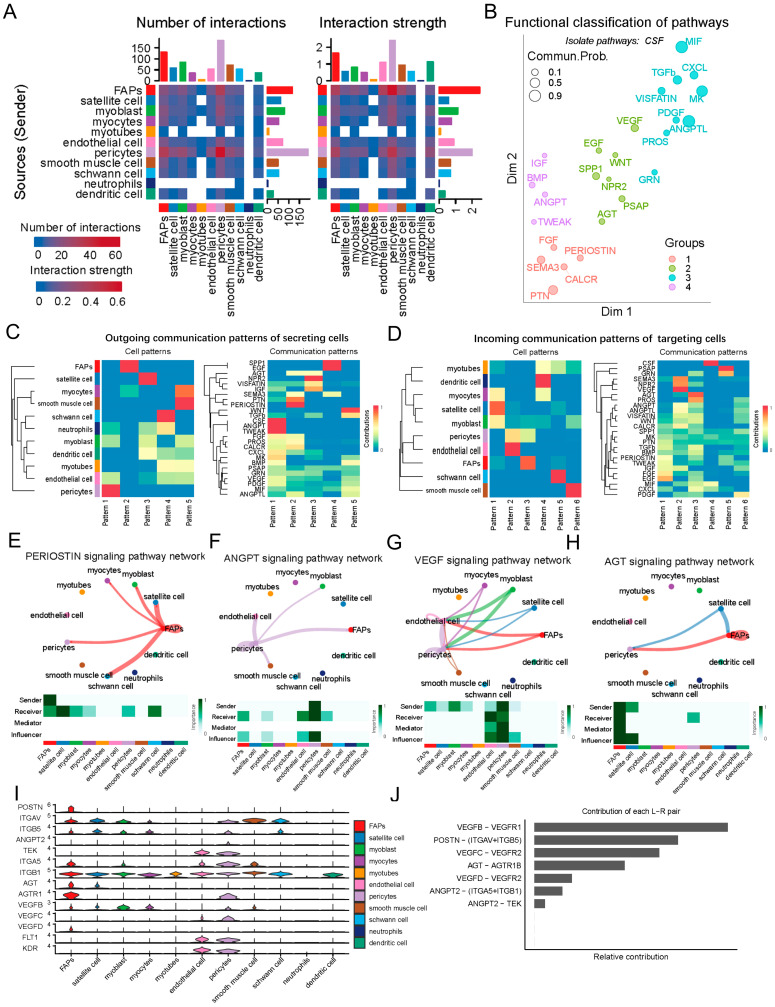
Communication network construction for the LD of the newborn Suhuai pig by CellChat. (**A**) The number of interaction plot of LD cells. The left graph is the number of interactions and the right graph is the interaction strength between different cell types. The top and right colored bar plots represent the sum of values displayed in the heatmap. (**B**) Projecting signaling pathways onto a two-dimensional manifold based on their functional similarity. Each dot represents the communication network of one signaling pathway. Dot size is proportional to the overall communication probability. Different colors represent different groups of signaling pathways. (**C**) Heatmap showing the outgoing communication patterns of secreting cells in LD. The relation between 5 inferred potential patterns with different cell populations (**left**) or signaling pathways (**right**) were shown. (**D**) Heatmap showing the incoming communication patterns of target cells in LD. The relation between 6 inferred potential patterns with different cell populations (**left**) or signaling pathways (**right**) were shown. (**E**–**H**) Communication network of 4 secretion (sender)/targeting (receiver) pathways in FAPs and pericytes, respectively. Each node (circle) represents a distinct cell type, while the connecting lines (edges) depict the known or hypothesized interactions. The thickness of the lines indicates the strength or frequency of interactions, and the different colors represent different types or subtypes of interactions. (**I**) The expression distribution of four signaling pathway related genes in different cell type in LD, including PERIOSTIN pathway (POSTN, ITGAV, ITGB5), ANGPT pathway (ANGPT2, TEK, ITGA5, ITGB1), AGT pathway (AGT, AGTR1), and VEGF pathway (VEGFB, VEGFC, VEGFD, FLT1, KDR). (**J**) Relative contribution of each ligand–receptor pair to the overall signaling network in LD.

After identification of cell types within the LD of the newborn Suhuai pig, we delved deeper into the intricate communications among various cell populations and the signaling pathways coordinating their functions. Utilizing CellChat, a method that employs pattern recognition based on nonnegative matrix factorization, we were able to decipher global communication patterns and key signals among different cell groups [21]. Our analysis detected 87 significant ligand–receptor pairs distributed across the 11 cell populations. These pairs were subsequently categorized into 26 signaling pathways, encompassing TGFβ, WNT, EGF, FGF, SPP1, PTN, PDGF, CXCL, VISFATIN, MIF, etc. Intriguingly, our findings revealed that FAPs and pericytes predominantly function within these signal networks, serving as the primary signal secretors/senders and targets/receivers, respectively (Figure 2A and Figure S2A and Supplementary Table S3).

Given the intricate nature of the communication networks within the LD of the newborn Suhuai pig, we inferred these networks by relying on their functional similarities. As a result, this approach enabled the identification of four distinct groups of pathways. As shown in Figure 2B, each group was enriched for a number of representatives signaling pathways; specifically, groups #1 and #4 were primarily characterized by pathways associated with osteogenesis (PERIOSTIN) and angiogenesis (ANGPT), respectively. Groups #2 and #3 predominantly encompassed pathways linked to myogenesis (WNT, NPR2, EGF) and immune response (MIF, VISFATIN), respectively. In contrast, CSF signal was isolated individually. Briefly, our findings indicate that the signaling within the communication network in LD of newborn Suhuai pigs predominantly involves processes related to osteogenesis, myogenesis, angiogenesis, and immune response.To elucidate the coordination between various cell groups and signaling pathways, we applied communication pattern analysis and revealed five distinct patterns for outgoing signaling (Figure 2C), and six patterns for incoming signaling (Figure 2D). The results showed that outgoing signaling from FAPs and myocytes was characterized by pattern 2, encompassing pathways PERIOSTIN, PTN, SEMA3 and AGT, etc. Furthermore, outgoing signaling from pericytes, endothelial cells, myoblasts, and neutrophils was characterized by pattern 1, which included pathways CSF, ANGPT, TWEAK, etc. (Figure 2C). Regarding incoming signaling, FAPs, pericytes and myoblast were characterized by pattern 3, encompassing pathways AGT, PROS, etc. (Figure 2D). The endothelial cells and pericytes were characterized by pattern 2, encompassing pathways VEGF, NRP2, SEMA3, etc. (Figure 2D).

Network centrality analysis of the PERIOSTIN signaling pathway network revealed that FAPs predominantly transmit signals to FAPs itself, satellite cells, pericytes, and Schwann cells via ligand–receptor pairs, specifically *POSTN-ITGAV/ITGB5* (Figure 2E,I,J). This result is consistent with the finding that FAPs are the principal initiators in driving bone cell migration, proliferation, and differentiation [22]. ANGPT2 plays a pivotal role in regulating the integrity of endothelial cell–cell junctions [23], and we found ANGPT signaling pathway network predominantly secreted by pericytes (Figure 2I), impacts itself, endothelial cells, FAPs, smooth muscle cells, and myoblasts through the ligand–receptor pairs *ANGPT2-ITGA5/ITGB1/TEK* (Figure 2F–J). VEGF, a crucial signal for vasculogenesis, synergistically promotes endothelial cell growth, migration, and vascular formation [24]. We found VEGF signaling is released by multiple cells and impacted pericytes, endothelial cells, smooth muscle cells, FAPs, satellite cells, myoblasts and myocytes through ligand–receptor pairs, including *VEGFB-VEGFR1*, *VEGFC-VEGFR2*, or *VEGFD-VEGFR2* (Figure 2G,I,J). Previous studies indicate that AGT signaling pathway primarily functions in maintaining blood pressure, fluid and electrolyte homeostasis, and promoting adipocyte differentiation [25,26]. As signal senders of AGT, we found that FAPs and satellite cells act on FAPs, and pericytes via ligand-receptor pairs *AGT-AGTR1B* (Figure 2H–J).

Overall, we systematically constructed communication networks for LD of the newborn Suhuai pig. Significantly, our findings highlight that FAPs and pericytes are the principal signaling contributors in LD of Suhuai pigs. This observation strongly suggests that FAPs and pericytes play pivotal roles in regulating the growth and developmental processes of LD.

### 2.3. Pseudotime Patterns Reveal the Relationships among Different Myogenic Populations

**Figure 3:**
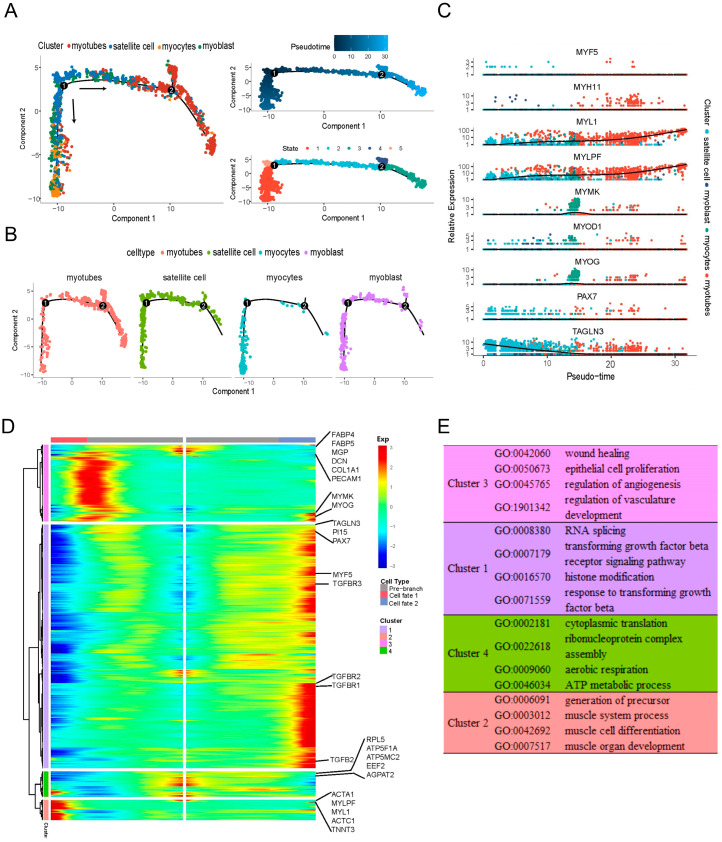
Pseudotime patterns of myogenic cell differentiation. (**A**) The pseudotime analysis result of satellite cells, myoblasts, myocytes and myotubes with monocle2. The 1 and 2 in the circle represent branch point 1 and branch point 2, respectively. Black arrows indicated two pseudo-direction of differentiation at branch point 1. (**B**) Distribution of each type of cell across pseudotime trajectory. (**C**) Scatter plot the expression levels of 9 marker genes across pseudotime. The *x*-axis represents pseudotime, the *y*-axis indicates the normalized gene expression levels, the colors refer to the four myogenic cell populations. (**D**) The differentially expressed genes (rows) of myogenic cells were clustered hierarchically into four groups, across the two pseudo-direction of differentiation at branch point 1. (**E**) The representative gene functions and pathways of each cluster were shown (Supplementary Table S5).

To estimate the lineage relationships of the myogenic cell populations in LD of newborn Suhuai pigs, we performed a pseudotime analysis of obtained scRNA-seq data focusing on myogenic-related cell populations, namely satellite cells, myoblasts, myocytes, and myotubes. We found that these four cell types underwent two distinct differentiation trajectories at branch point 1 and point 2, respectively (Figure 3A,B). In terms of the two differentiation directions at branch point 1, we found that for one direction, the progression was from satellite cells to myoblasts, myocytes, and finally myotubes. The other direction followed a path from satellite cells to myoblasts and myotubes, excluding the formation of myocytes. In addition, the two differentiation directions at branch point 2 predominantly occurred in myotubes (Figure 3A,B). Next, the expression trends for nine marker genes of these four cell type were plotted across pseudotime (Figure 3C). We found that *PAX7* and *MYF5* were mainly expressed in satellite cells, marking the early stage of pseudotime. *MYOD1*, *MYOG*, and *MYMK* genes were predominantly expressed in myocytes, constituting the midpoint of pseudotime. *MYH11* gene was highly expressed in myotubes, marking the late stage of pseudotime. Notably, *TANGLN3* exhibited a decreasing expression trend across pseudotime, in contrast to the increasing trends observed for *MYL1* and *MYLPF*.

Next, we further plotted the differentially expressed genes across the two pseudotime trajectories at branch point 1. We observed four distinct gene expression clusters (Figure 3D), including 2511, 206, 790, and 261 genes, respectively (Supplementary Table S4). As shown in Figure 3D,E and Supplementary Table S5, genes in cluster 1, such as TAGLN3, *PI15*, *PAX7*, *MYF5* and *TGFb*-related genes exhibited high expression levels at the terminal stage of cell fate 2 branch, but low expression in cell fate 1 branch. This cluster 1 genes were predominantly enriched GO terms of RNA splicing, transforming growth factor beta receptor signaling pathway, histone modification, and response to transforming growth factor beta, etc. (Figure 3E). Conversely, genes in cluster 2, such as *ACTA1*, *MYLPF*, *MYL1*, *ACTC1*, and *TNNT3*, were identified with high expression at the terminal stage of cell fate 1 branch, but low expression in cell fate 2 branch. This cluster 2 genes were associated with GO terms such as generation of precursor, muscle system process, muscle cell differentiation, and muscle organ development, etc. (Figure 3E). Cluster 3, mostly expressed at the early and the junction stages of cell fate 1 branch, related to wound healing, epithelial cell proliferation, regulation of angiogenesis, and vasculature development, etc. (Figure 3E). Remarkably, most of genes in cluster 4 displayed a decreasing expression trend from the pre-branch stage to two different cell fates, and this cluster genes were linked to functions in cytoplasmic translation and ATP metabolic processes, etc. (Figure 3E). These results suggested that the two differentiation fates of myogenic cells at branch point 1 exhibited distinct gene expression signatures.

Overall, these analyses delineated the pseudotime differentiation trajectory underlying myogenesis in LD of newborn Suhuai pigs, revealing that myogenesis is a heterogeneous cellular process occurring at various temporal stages.

### 2.4. FAPs Were Associated with the Differentiation of Tendon

**Figure 4:**
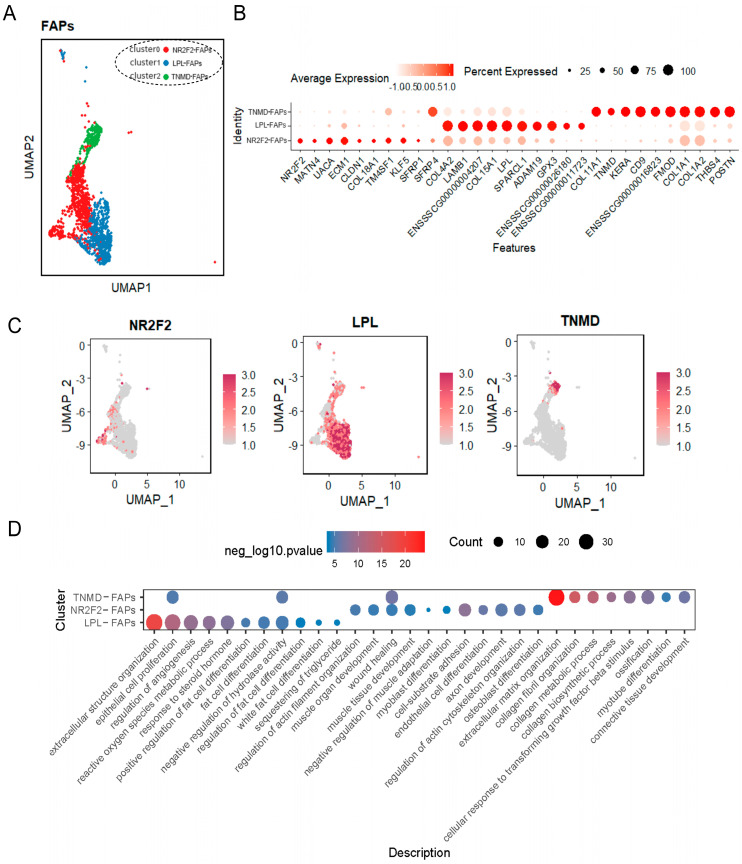
Heterogeneity analysis of sub-clusters of FAPs. (**A**) FAPs were divided into three sub–clusters. (**B**) Expression of top 10 differential expressed genes in three sub-clusters from Supplementary Table S6. (**C**) The feature plots showed three sub-clusters of FAP–expressing specific genes. (**D**) Selected GOs enriched in three sub-clusters. Dot size corresponds to the count value of a process. The size of dot corresponds to the count value for certain GO. The color depth of dot corresponds to the negative log10 *p*–value for certain GO.

In meat production, a high content of intramuscular fat is positively correlated with meat quality traits [27]. Recent studies have shown that FAPs contribute significantly to intramuscular fat formation [28,29]. And, FAPs have the potential ability to differentiate into myofibers, lipogenesis and osteogenesis [22,30]. Therefore, we performed subclustering analysis of FAPs to delineate its multi-directional differentiation potential. The clustering analysis divided FAPs into three sub-clusters (Figure 4A and Supplementary Table S6), and the top 10 differential genes of each cluster were displayed in Figure 4B. Specifically, cluster 0 was characterized by the high expression of genes such as *NR2F2*, *MATN4*, *UACA*, *CLDN1*, etc. Cluster 1 characterized by the expression of genes including *LPL, LAMB1*, *ADAM19*, *GPX3*, etc. Cluster 2 highly expressed several genes associated with collagen fiber formation, tendonogenesis, and angiogenesis, namely *COL11A1*, *TNMD*, *FMOD*, *COL1A1*, *COL1A2*, etc. (Figure 4B). Combining the biological properties and gene expression characteristics of FAPs, we tentatively name these three sub-clusters as *NR2F2*-FAPs (cluster 0), *LPL*-FAPs (cluster 1), and *TNMD*-FAPs (cluster2) (Figure 4A,C). Notably, cluster 2 was highly specific for the expression of *TNMD*, which has been shown to be a marker gene for tendon cells [31,32,33].

To further reveal the heterogeneity among sub-clusters of FAPs. We investigated the heterogeneity of cellular subpopulations of FAPs. GO-terms of enrichment enriched 386 terms in total, and these three sub-clusters enriched distinct GOs, respectively (Figure 4D and Supplementary Table S7). For instance, *LPL*-FAPs related to fat cell differentiation, response to steroid hormone, white fat cell differentiation, etc.; *NR2F2*-FAPs related to muscle tissue development, myoblast differentiation, regulation of actin cytoskeleton organization, etc.; and *TNMD*-FAPs related to wound healing, connective tissue development, collagen metabolic process, ossification, etc.

In conclusion, these results revealed that FAPs are involved in multiple physiological processes such as lipogenesis, osteogenesis, wound healing, and angiogenesis [22,34,35]. Intriguingly, our findings imply that tendon development is linked to FAPs, which provides new insight for the physiological function of FAPs.

## 3. Discussion

The LD of pigs is a representative muscle model for lean meat production and meat quality assessment in pig breeding. Using single-cell analysis allows us to explore the identity of individual cells, cell–cell interaction, and differentiation trajectories. Here, we constructed a cellular atlas and signaling network for LD muscle in the newborn Suhuai pig, further estimated the lineage relationships among myogenic cell populations, and revealed the heterogeneity of FAP population. In total, our scRNA-seq data identified 11 cell populations using a strictly filtered mononuclear cell suspension isolated from LD muscle of a 1-day-old newborn Suhuai boar (Refer to Materials and Methods). It is known that scRNA-seq technology was unable to capture multinucleated myotubes [36]; however, we identified 18.82% myotubes in this study, suggesting that these myotubes should be mononucleated cells. Since these mononucleated myotubes expressed the marker genes of multinucleated myotubes, such as *CKM*, *TNNT3*, *MYLPF*, *MYL1*, and *ACTA1* [37], indicating that the *MYOD1* and *MYOG* positive myocytes first initiated the process to express the terminal differentiation above mentioned marker genes to form the mononucleated myotubes, and then fused to multinucleated myotubes in the late stage of myogenesis.

A recent elegant work has profiled LD of three pig breeds using scRNA-seq [6]. Theoretically, heterogeneity could exist in the LD cell atlases in different pig breeds. For instance, using the well-documented marker genes (*CD34*, *PECAM1*, and *CDH5*) [38,39], we found the proportion of endothelial cells was the largest (24.39%) in our work, which is quite different from the recent study (less than 1% for all three pig breeds) [6].

Although numerous studies have identified specific markers for individual cell types, it is noteworthy that the expression levels of these markers can vary across different physiological stages. In our work, we observed low expression levels of *MYF5* in satellite cells, which acts as a myogenic factor to promote satellite cell proliferation [30]. Similarly, Xu et al. found that *MYF5* was barely expressed in satellite cells of neonatal Duroc pigs, Laiwu pigs, and wild boar LDs [6]. Differently, *MYF5* is highly expressed in myogenic progenitors/myoblasts in pig embryos [5]. These findings indicates that *MYF5* may not play a significant role in myogenesis during the newborn stage.

Pericytes, also referred to as mural cells, envelop capillary blood vessels on their abluminal side. Structurally, these cells extend from their bodies, covering multiple endothelial cells [40]. The ability of pericytes to differentiate into endothelial cells is a topic of research and debate in the field of vascular biology. In this study, we observed that the endothelial cell marker genes were highly expressed in pericytes, which is consistent with one previous finding from skeletal muscle biopsies using scRNA-seq [41], and further corroborated that pericytes are related to the differentiation of endothelial cells. In addition, multiple pericyte markers have been identified (such as *EBF1*, *COX4I2* [42], *RGS5* [43], and *PDGFRB* [44]). However, we found these pericyte markers are highly expressed in smooth muscle cells as well. These results suggest that the expression of pericyte markers is complicated, and may vary depending on developmental stages, physiological condition, and other factors [45]. Therefore, further investigation is needed to better understand pericyte marker genes more deeply.

In addition, we found that genes associated with mitosis (such as *TOP2A, PCNA* and *NUSAP1*) and cell cycle regulation (such as *E2F* family transcription factors) were highly and specifically expressed in myoblasts. *E2F* family genes are critical, as the entry of satellite cells into the cell cycle from quiescence required the activation of the Cdk/Rb/E2F signaling pathway, because *E2F* regulates genes involved in DNA replication and cell cycle progression [46,47,48]. Our data confirmed that myoblasts we identified in LD were activated satellite cells, which was on the way to differentiate into myocytes and myotubes.

The construction of cell–cell communication networks using the expression levels of ligand–receptor genes can yield significant biological insights. In our work, the cellular communication networks we constructed reveal unique modes of regulation for different cell types in the LD of newborn pig. The complex signaling network was categorized into four distinct groups based on functional similarity of signaling pathway, including osteogenesis, myogenesis, angiogenesis, and inflammation. This categorization greatly simplified the amount of information within the communication network. Interestingly, the CSF signal emerged as an isolated entity, reflective of its unique physiological function within the network. This finding aligns with the expectation that muscle and vascular developmental processes are particularly prominent in newborn muscle tissues [49]. Additionally, the synergistic effects of signal outgoing and incoming between cells were investigated through the analysis of receptor or ligand expression patterns in multicellular populations. Notably, although the number of pericytes was less than 2%, pericytes and FAPs showed the strongest intercellular relationships among multiple cell populations. These results are consistent with the recent study [6]. We speculated that this might be associated with the physiological characteristics inherent to the neonatal stage of pigs.

The pseudotime analysis showed that four myogenic cell types underwent two branch points. The reasons for first branch differentiation are unclear, since it does not make sense that the myoblasts can directly differentiate into myotubes without undergoing the formation of *MYOD1* and *MYOG* positive myocytes. However, the myotubes formed in this differentiation direction is very interesting, because they underwent the second branch differentiation (Figure 3A, branch point 2), possibly formed different types of muscle fibers. However, further studies are needed to elucidate the differentiate trajectory of myogenic cells more thoroughly.

It is well-known that FAPs can differentiate into fibroblasts, adipocytes, and osteoblasts/chondrocytes [50]. In this study, the heterogeneity within FAP sub-clusters also revealed its multi-directional differentiation potentials. It is noteworthy that we found *TNMD*, a marker gene for tendon cells [31,32,33], is specifically expressed in one FAP subpopulation (*TNMD*-FAPs), and this finding is consistent with the recent study [6]. Previous research demonstrated the differentiation of *TNMD* expressed tendon cells derived from the tendon progenitor cells [51,52]. This study further suggested that tendon progenitor cells could differentiate into osteoblasts, chondrocytes, or adipocytes [52]. In addition, chondrogenesis has been observed in adult tendon regeneration [53]. *TGF-β* has also been reported to effectively promote the formation of tendons and ligaments through interaction with *Sox9*, a transcription factor of cartilage formation [54]. These pieces of evidence suggest that tendon progenitor cells shared similar physiological functionally as to FAPs. We speculated that FAPs may be closely related to tendon cell differentiation. However, Scleraxis (Scx), as one known marker expressed in tendon progenitor [55], was not enriched in identified FAPs at present study. This discrepancy needs further investigation.

In conclusion, we constructed a cellular atlas and communication network of the LD of the newborn Suhuai pig, and revealed heterogeneous cellular processes in muscle developmental events. Meanwhile, we have discovered a strong association between tendonogenesis and FAPs. The limitation of this study is that the relationship between FAPs and tendonogenesis needs further investigation. Overall, this work provides a useful resource for the study of skeletal muscle development and meat quality control in pigs.

## 4. Materials and Methods

### 4.1. Animal and Sample Collection

The experimental animal was handled according to Guidelines for the Care and Use of Laboratory Animals prepared by the Institutional Animal Welfare and Ethics Committee of Nanjing Agricultural University, Nanjing, China. The 1-day-old newborn Suhuai boar (purchased from Huaiyin Pig Breeding Farm of Huaian City) was anesthetized and euthanized. Then, the skin of the back was disinfected, and the LD was taken for cell suspension preparation using a sterile scalpel (1 cm^3^).

### 4.2. Preparation of Single-Cell Suspensions

The collected LD sample was washed three times with PBS, then appropriate amount of PBS was added and the sample was cut with ophthalmic scissors (<1 mm). Then, the sample was digested using 4 mg/mL papain (Sigma-Aldrich (St. Louis, MO, USA) cat# 9001-73-4), 20 mg/mL collagenase II (Sigma-Aldrich, cat# 9001-12-1), and 20 mg/mL collagenase IV (Sigma-Aldrich, sku# C4-22-1G). The digestion reactions were shaken vigorously for 30 s and further incubated at 37 °C for 30 min in an incubator with general shaking every 6 min to release cells. The released cells were consecutively passed through 100 μm, 70 μm, and 40 μm cell strainer (BD, 352350) and collected the mononuclear cells in 15-mL tubes. Sample viability was assessed via Trypan Blue (Thermo Fisher (Waltham, MA, USA)) and automatic cell counter (Countstar (Shanghai, China)).

### 4.3. Generating the 10× Genomics ScRNA-Seq Library and Sequencing

Droplet-based scRNA-seq datasets were produced using a Chromium system (10× Genomics, PN120263) following the manufacturer’s instructions. Single-cell suspensions were quality checked and counted, requiring a survival rate of 80% or more and a cell concentration of 700–1200 cells/µL. Gel Beads in Emulsion (GEMs) were then constructed for single-cell isolation and reverse transcribed in a PCR machine to construct RNA libraries. After passing quality control, double-end sequencing was performed on the Illumina NovaSeq-6000 platform using PE150.

### 4.4. Sequencing Data Quality Control

After sequencing, a total of around 120 Gb raw data and 389,705,520 reads were obtained. Sequencing reads were processed and analyzed using 10× Genomics Cell Ranger 3.0.1 software, which consists of different analysis pipelines [56]. The Sscrofa11.1 reference genome was downloaded from the Ensemble. A matrix with barcodes and gene expression counts was generated by calculating unique molecular identifiers (UMIs) and filtering for noncell-related barcodes. The raw digital gene expression matrix (number of UMIs per gene per cell) was filtered, normalized and clustered using R (v3.5.2, https://www.R-project.org, accessed on 1 December 2021). Cells were only retained if the number of detected genes was greater than 500 and less than 6000, and the percentage of detected transcripts from mitochondrial genes (*ATP6*, *ATP8*, *COX1*, *COX2*, *COX3*, *CYTB*, *ND2*, *ND3*, *ND4*, *ND4L*, *ND5*, *ND6*) was less than 12%.

### 4.5. Cell Clustering and Cell Type Annotation

Normalization was performed in the Seurat R package (v3.1.1) using the default parameters [57]. After principal component analysis (PCA), the first 13 principal components were selected for clustering the cells using standard package procedures. The Louvain algorithm with a resolution of 0.4 was used to cluster cells. A gene was considered to be differentially expressed if it was detected in at least 10% of one group and with at least 0.25 log fold change between two groups, and a Benjamini–Hochberg (BH) adjusted *p* value < 0.05 in Wilcoxon rank-sum test was considered to indicate significance. Cell clusters were annotated based on recently reported marker genes and significantly enriched expression (with B-H adjusted *p* value below 0.05) of marker genes of different cell types. To investigate the heterogeneity within FAPs, we conducted subclustering analysis of FAPs using recently reported marker genes and specifically expressed genes.

### 4.6. Analysis of Cellular Communication Networks

CellChat [21], an open-source R package (https://github.com/sqjin/CellChat, accessed on 1 July 2022), was used to infer, visualize and analyze intercellular communication for scRNA-seq data. Briefly, after identifying the database, CellChat infers cell-state-specific signaling communications within a given scRNA-seq dataset using mass action models, along with differential expression analysis and statistical tests on cell groups, which can be either discrete states or continuous states along the pseudotime cell trajectory.

### 4.7. Trajectory Analysis of Myogenic Cell

To construct the pseudo-temporal path of myogenic cell differentiation, the Monocle algorithm (version 2.20.0) [58] was used. The reduceDimension and orderCells functions of the DDRTree method were utilized to order the myogenic cells along the pseudotime trajectory. Based on the expression patterns of the genes, hierarchical clustering was used to classify the genes into four subclusters. Heatmap of gene expression following pseudotime was plotted using the “plot-pseudotime-heatmap” function. The gene expression curves were calculated and plotted based on the average value of gene expression for each unit of pseudotime. The *x*-axis, labeled as pseudotime, represents the transformed values derived from the original pseudotime values of cells in time trajectory analysis. These values are mapped onto a standardized interval ranging from 0 to 100, providing a simplified and uniform scale for evaluation and comparison within the dataset. Gene expression changes at branch point 1 were then analyzed using the special branched expression analysis modelling (BEAM) statistical test provided by Monocle2.

### 4.8. Gene Ontology (GO) Enrichment Analysis

Gene Ontology (GO) analysis was performed using the clusterProfiler 4.0 package [59]. Because of the lack of the relative studies in pigs, the GO terms of selected genes were enriched in the database “org.Hs.eg.db” using the “enrichGO” function. The Benjamini–Hochberg (BH) method was used for the multiple test adjustment.

## Data Availability

The single-cell RNA sequencing data used in this research was deposited in in GEO (https://www.ncbi.nlm.nih.gov/geo/query/acc.cgi?acc=GSE247753, accessed on 14 November 2023) with the accession number: GSE247753 (release date: 1 June 2024).

## Supplementary Materials

The following supporting information can be downloaded at: https://www.mdpi.com/article/10.3390/ijms25021204/s1.
