# Supplementary material for: Single-Cell RNA Sequencing Reveals the Cellular Landscape of Longissimus Dorsi in a Newborn Suhuai Pig
Source: Int J Mol Sci. 2024 Jan 18;25(2):1204. doi: 10.3390/ijms25021204 (PMC10816681; doi:10.3390/ijms25021204)
Supplement: Supplementary file 1 [file ijms-25-01204-s001.zip › Supplementary Figures S1 and S2.pdf]

Supplementary Materials

Single-Cell RNA Sequencing Reveals the Cellular Landscape of *Longissimus Dorsi* in a Newborn Suhuai Pig

Wei Xiao [1,2,3], Nengjing Jiang [1,2,3], Zhengyu Ji [1,2,3], Mengru Ni [1,2,3], Zhaobo Zhang [1,2], Qingbo Zhao [1,2,3,4], Ruihua Huang [1,2,3,4], Pinghua Li [1,2,3,4] and Liming Hou [1,2,3,4,*]

[1] College of Animal Science and Technology, Nanjing Agricultural University, Nanjing 210095, China; weifortunately@gmail.com (W.X.); 18751997986@njau.edu.cn (N.J.); jzy604672583@gmail.com (Z.J.); nmr1999@163.com (M.N.); zhaobo1312@126.com (Z.Z.); zhaoqingbo@njau.edu.cn (Q.Z.); rhhuang@njau.edu.cn (R.H.); lipinghua718@njau.edu.cn (P.L.)

[2] Institute of Swine Science, Nanjing Agricultural University, Nanjing 210095, China

[3] Key Laboratory of Pig Genetic Resources Evaluation and Utilization (Nanjing) of Ministry of Agriculture and Rural Affairs, Nanjing Agricultural University, Nanjing 210095, China

[4] Huai'an Academy, Nanjing Agricultural University, Huai'an 223001, China

* Correspondence: liminghou@njau.edu.cn or mnhouliming@126.com

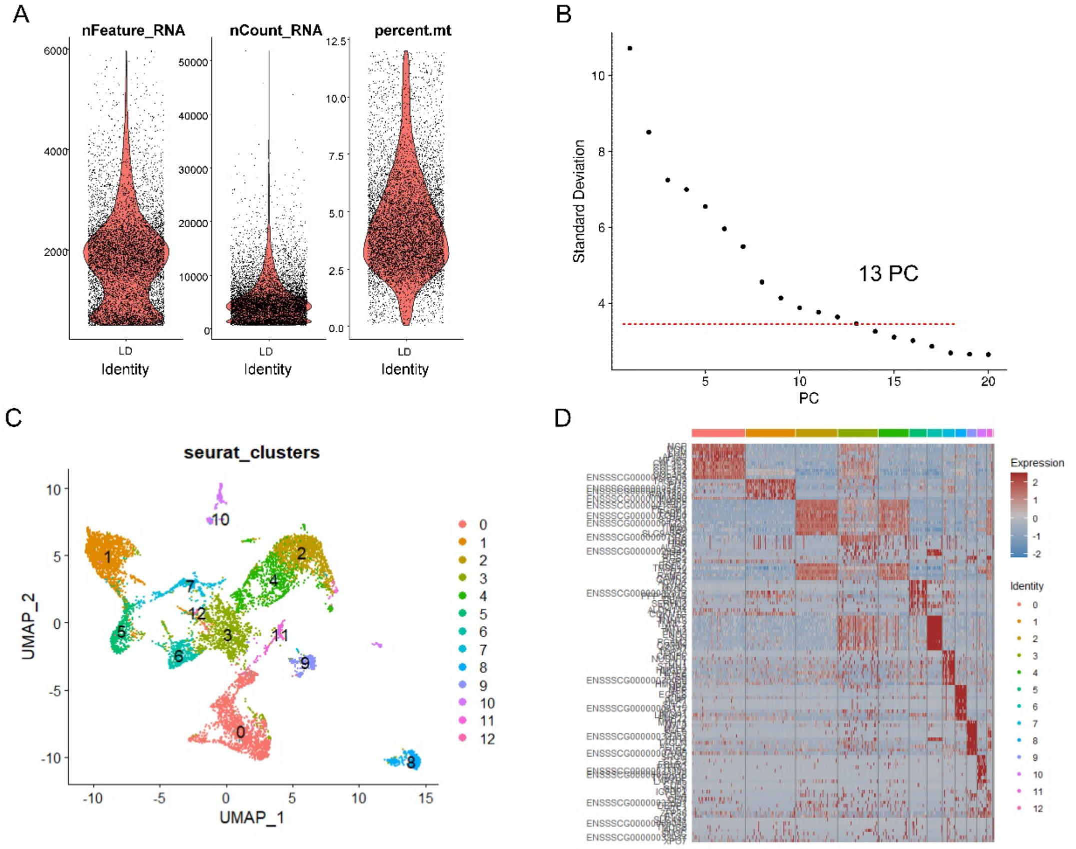

Figure S1. Data quality control and principal component analysis. (**A**) Violin diagram showing the number of genes (nFeature), the number of unique molecular identifiers (UMIs) (nCount), and the distribution ratio of mitochondrial genes (percent. MT) data quality control. (**B**) Elbow plot showing standard deviations of principal components (PCs). The x-axis represents the PCs, the y-axis represents their standard deviations. The 'elbow' at PC 13, in where the decline of standard deviation slows, indicating a natural cut-off for significant components. (**C**) UMAP plot of LD cells (N = 9508) identified in LD of newborn Suhuai boar. Each dot represents one cell. Dot color corresponds to cell type annotation. (**D**) Heatmap exhibited distinct expression pattern of top 10 expressed genes for each cluster. The expression level of each gene from low to high is indicated by a color gradient from steel blue to red.

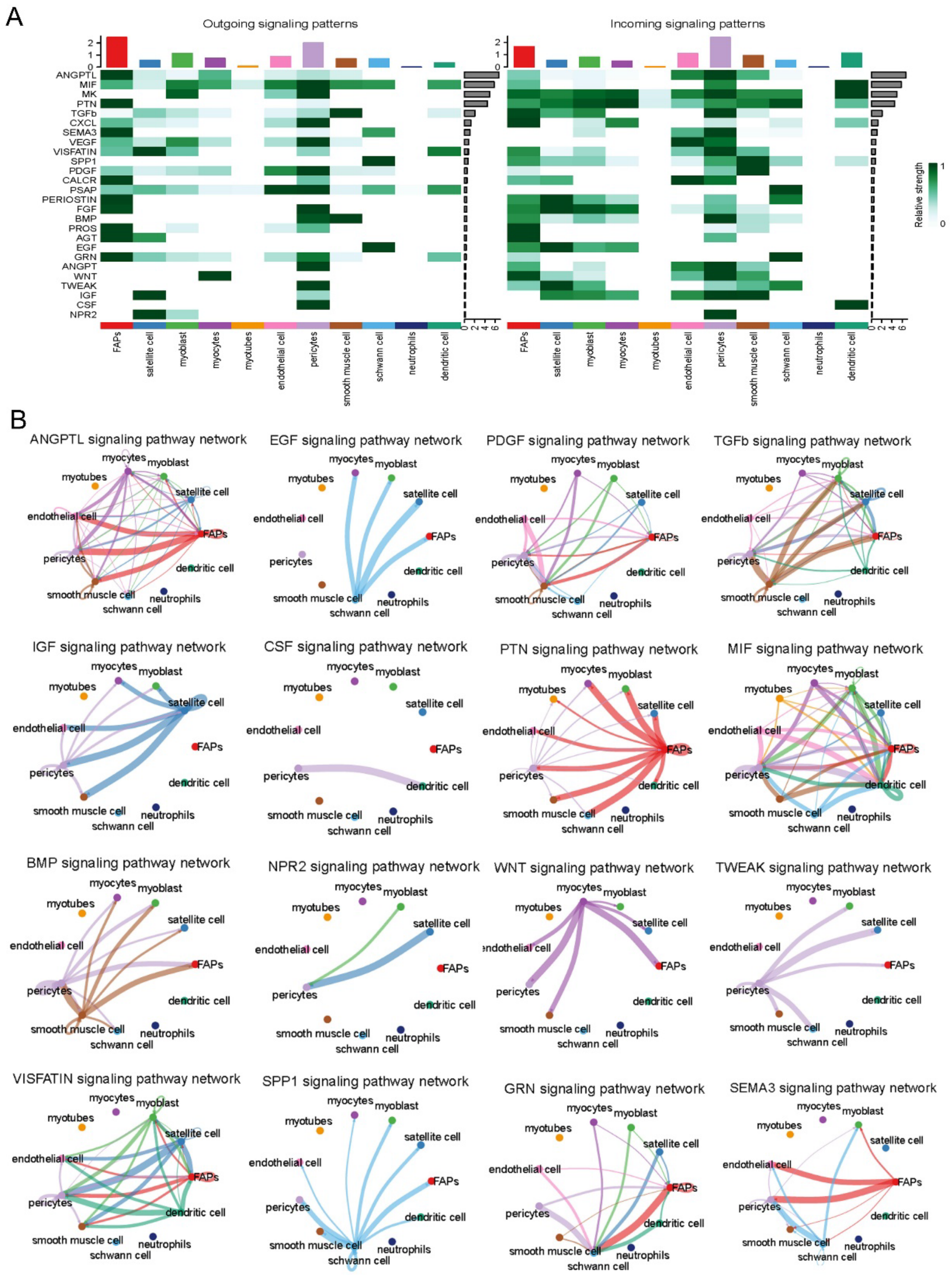

Figure S2. Communication network construction for LD of newborn Suhuai pig by CellChat. (**A**) Heatmaps of all signal pathways contributing mostly to outgoing or incoming signaling of certain cell groups. The top-colored bar plot represents the sum of value displayed in the heatmap. Dark green (or white) colored edges represent increased (or decreased) signaling. The bar plot on the right indicates the contribution of the signaling pathways. (**B**) The network diagrams showed the signaling pathways between different cell populations, including classical growth factor signaling pathways (ANGPTL, EGF, PDGF, TGFb, IGF, CSF, and PTN), angiogenesis (VEGF, NPR2), osteoblast differentiation (BMP, PERIOSTIN), muscle development (WNT, MIF), and immune related pathways (VISFATIN, GRN, SEMA3). Each node (circle) represents a distinct cell type, while the connecting lines (edges) depict the known or hypothesized interactions. The thickness of the lines indicates the strength or frequency of interactions, and the different colors represent different types or subtypes of interactions.
